# In this issue: Emerging and provisional B and T cell lymphoma entities

**DOI:** 10.1007/s00428-023-03597-4

**Published:** 2023-09-29

**Authors:** Leticia Quintanilla-Martinez

**Affiliations:** Eberhard-Karls Universität, Universitätsklinikum Tübingen, Tubingen, Germany

This month’s issue includes a mini-series of 5 review papers on “Emerging and provisional B and T cell lymphoma entities.” These review papers resulted from the 2022 lymphoma workshop of the European Association for Haematopathology/Society for Hematopathology held in Florence, Italy.

After the 4th revised World Health Organization (WHO) classification was published, much has been learned about the genetic landscape of lymphomas, as a consequence of new techniques used for routine diagnosis, and in translational and basic research. In the last years, we have witnessed a growing list of genetic aberrations that have helped to define new disease entities, as well as understanding the pathogenesis of certain lymphomas. The identification of recurrent *IRF4* rearrangements in a group of lymphomas previously considered part of pediatric-type follicular lymphoma helped to delineate the morphological and molecular spectrum of large B-cell lymphoma with *IRF4* rearrangement (LBCL-*IRF4*). The recurrent gains and telomeric losses of chromosome 11 became the genetic alteration of a new entity affecting mostly children and young adults with morphology and phenotype similar to Burkitt lymphoma but without *MYC* translocation named high-grade/large B-cell lymphoma with 11 q aberration (HG/LBCL-11q). Similarly, as in diffuse large B-cell lymphomas (DLBCL), gene expression profiling in T-cell lymphomas has been translated into immunohistochemical algorithms to facilitate the diagnosis of peripheral T cell lymphomas, not otherwise specified (PTCL, NOS). This algorithm needs to be validated. Mutational analysis is demonstrating that gene mutations considered part of clonal hematopoiesis (*TET2* and *DNMT3A*) are present not only in follicular helper T-cell (TFH) lymphomas but also in cytotoxic PTCL, NOS. Furthermore, the association between TFH proliferations and cytotoxic PTCL, NOS seems to be more frequently than previously thought. Nevertheless, despite all the advances with new molecular techniques, some old challenges persist. The differential diagnosis between TFH lymphomas and TFH reactive lymphoproliferations is not always easy to resolve. The diagnostic criteria for HHV8-negative, EBV-negative effusion lymphomas need to be clarified. The border between some of the HHV8-positive lymphomas is not always easy, especially between HHV8-positive DLBCL and extra-cavitary primary effusion lymphomas (ECPEL). The diagnostic criteria of marginal cell lymphomas have evolved and the underlying mechanism responsible for its pathogenesis are started to be elucidated, although much work needs to be done.

Another important event last year was the publication of two lymphoma classifications: the 2022 International consensus classification (ICC) and the 5th edition of the WHO. The articles within this series review different B- and T-cell lymphoma entities highlighting diagnostic challenges, new molecular mechanisms, unusual variants, useful criteria but also new terminology. It is important to stress that in some entities criteria and terminology differ between the two classifications. These differences are discussed and based on published data and the cases submitted to the workshop, recommendations are given on how to incorporate this information into our routine diagnosis. In general, it is recommended to use both classifications.

The reviews in this series include the following:

- Emerging entities: high-grade/large B cell lymphoma with 11q aberration, large lymphoma with *IRF4* rearrangements, and new molecular subgroups in large B-cell lymphomas.
- Cavity-based lymphomas: challenges and novel concepts.
- The many faces of nodal and splenic marginal zone lymphomas.
- Cytotoxic peripheral Tcell lymphomas and EBV-positive T/NK- cell lymphoproliferative diseases: emerging concepts, recent advances, and the role of clonal hematopoiesis.
- Follicular helper T-cell lymphomas: disease spectrum, relationship with clonal hematopoiesis, and mimics.

Quintanilla-Martinez et al. (10.1007/s00428-023-03590-x) review emerging entities and new molecular subgroups in LBCL. The manuscript is focused on newly recognized diseases and their diagnostic challenges including HG/LBCL-11q and LBCL-*IRF4* and others. Fluorescence in situ hybridization (FISH) analysis is recommended for the diagnosis of HG/LBCL-11q, a disease that predominates in children and young adults and can occur in the setting of immunodeficiency, including ataxia-telangiectasia. LBCL-*IRF4* occurs mainly in pediatric populations but also in adults with similar characteristics. It is usually an indolent disorder with excellent prognosis. *IRF4* rearrangement are demonstrated in most cases; however, cryptic translocations occur in approximately 10% of the cases. In the correct context the presence of IGH/IGK/IGL breaks and/or *IRF4* mutations in exon 2 support the diagnosis. The presence of *IRF4*-R associated with other chromosomal translocations (*BCL2, MYC, CCND1*) can be observed in a variety of aggressive B-cell lymphomas. These cases should not be diagnosed as LBCL-*IRF4*. Other novel molecular groups of LBCL are discussed, highlighting challenging features.

Di Napoli et al. (10.1007/s00428-023-03599-2) review cavity-based lymphomas and their challenges in their diagnosis and novel concepts. HHV8-associated lymphoproliferations are discussed. Primary effusion lymphoma (PEL) represents the prototype, which is usually diagnosed in the setting of human immunodeficiency virus (HIV)-positive individuals. Extra-cavitary PEL (ECPEL) is morphologically similar to PEL. The challenges in distinguishing ECPEL and HHV8-positive DLBCL are thoroughly discussed, especially the association with EBV. Another difficult area in the diagnosis of these rare diseases is the differential diagnosis between ECPEL and germinotropic lymphoproliferative disorder (GLPD). HHV8-negative effusion-based lymphomas need more precise criteria. However, EBV+ cases, usually associated with immunosuppression, and plasmablastic morphology/immunophenotype should be excluded. The fibrin-associated (FA)-DLBCL submitted demonstrated that there are *bona-fide* EBV-negative cases, and cases with plasmablastic morphology and immunophenotype, expanding the definition of this disease. FA-DLBCL can also present in breast implants and should be differentiated from breast implant-associated anaplastic large-cell lymphoma (BIA-ALCL).

Alberto Zamò et al. (10.1007/s00428-023-03633-3) review the challenging topic of splenic and marginal zone lymphomas. The difficulties in the differential diagnosis among splenic marginal zone lymphoma, splenic diffuse red pulp lymphoma, and hairy cell leukemia variant (splenic B cell lymphoma with prominent nucleoli) are highlighted, and the importance of integrating the clinical features, morphology, and immunophenotype in blood, bone marrow, and spleen. Another challenging topic discussed is the increased numbers of TFH cells in marginal zone lymphomas sometimes mimicking TFH lymphoma. Recommendations to help in the differential diagnosis are emphasized. The criteria of transformation in marginal zone lymphoma are unclear and a matter of continuous discussion. The overlapping features of pediatric nodal marginal zone lymphoma and pediatric-type follicular lymphoma are discussed, as well as the unifying concept under the name of pediatric-type follicular lymphoma with and without marginal zone lymphoma. The differential diagnosis with “atypical” marginal zone hyperplasia in children was underscored.

Climent et al. (10.1007/s00428-023-03616-4) review cytotoxic PTCL and EBV-positive T/NK cell LPD and the new concepts emerged from the workshop, highlighting the role of clonal hematopoiesis in the pathogenesis of these lymphomas. The new recognized entity primary nodal EBV+ T and NK cell lymphoma is thoroughly discussed. Criteria to distinguish this disorder from extra-nasal NK/T cell lymphoma, nasal type, systemic EBV+ T cell lymphoma of childhood, and aggressive NK cell leukemia are presented. Primary nodal EBV+ T/NK cell lymphoma is rare and occurs mostly in elderly individuals with underlying immune deficiency. By definition, these lymphomas involved primarily the lymph nodes but secondary involvement in extra-nodal sites are observed. The mutational landscape of this disease is similar to that of EBV-negative cytotoxic PTCL cases and is characterized by frequent mutations in *TET2* and/or *DNMT3A*, and JAK-STAT pathway genes, suggesting a potential role of clonal hematopoiesis in the pathogenesis of EBV-positive and -negative cytotoxic PTCL. The rather frequent association of cytotoxic PTCL, NOS, with TFH lymphoproliferations is also discussed.

Ondrejka et al. (10.1007/s00428-023-03607-5) review TFH lymphoma, its broad morphological spectrum, and unifying concept, as well as its relation with clonal hematopoiesis. TFH lymphomas include three morphological subtypes: angioimmunoblastic-type (AITL), which is the prototype of the disease, follicular type, and NOS. Although TFH lymphomas are considered aggressive lymphomas, unusual cases with early or indolent presentations are recognized. Other features like association with B cell proliferation, presence of Hodgkin/Reed-Sternberg cells, or the relationship between TFH lymphomas with clonal hematopoiesis and myeloid neoplasms are highlighted. The differential diagnosis between TFH lymphomas and reactive and neoplastic mimics is discussed, especially with the newly recognized variant of nodal marginal zone lymphoma rich in TFH cells. Molecular analysis is necessary in many cases and highlights the contribution of new technologies to the current understanding and classification of TFH lymphomas. Novel gene mutations in lymphoepithelioid/Lennert-like lymphoma are described.

The reviews presented here highlight the relevance of the lymphoma workshops with the systematic expert review of typical and unusual cases giving the unique opportunity to generate new knowledge and refine diagnostic criteria. At the same time new questions are raised that foster future investigations to improve our classification system for the benefit of the patients.

Leticia Quintanilla-Martinez

Review Editor Virchows Archiv

Guest editor of the mini-series

Institute of Pathology, University Hospital Tübingen

Eberhard Karls University of Tübingen

Tübingen, Germany

